# Challenges and Opportunities for Companies to Build HTA/Payer Perspectives Into Drug Development Through the Use of a Dynamic Target Product Profile

**DOI:** 10.3389/fphar.2022.948161

**Published:** 2022-07-18

**Authors:** Ting Wang, Neil McAuslane, Wim G. Goettsch, Hubert G. M. Leufkens, Marie L. De Bruin

**Affiliations:** ^1^ Centre for Innovation in Regulatory Science (CIRS), London, United Kingdom; ^2^ Division of Pharmacoepidemiology and Clinical Pharmacology, Utrecht Institute for Pharmaceutical Sciences, Utrecht University, Utrecht, Netherlands; ^3^ National Health Care Institute, Diemen, Netherlands

**Keywords:** drug development, evidence generation, health technology assessment (HTA), target product profile (TPP), reimbursement, company strategies, value proposition

## Abstract

**Background:** The target product profile (TPP) outlines the desired profile of a target product aimed at a particular disease and is used by companies to plan clinical development. Considering the increasing importance of health technology assessment (HTA) in informing reimbursement decisions, a robust TPP needs to be built to address HTA needs, to guide an integrated evidence generation plan that will support HTA submissions. This study assessed current practices and experiences of companies in building HTA considerations into TPP development.

**Methods:** An opinion survey was designed and conducted in 2019, as a cross-sectional questionnaire consisting of multiple-choice questions. The questionnaire provided a qualitative assessment of companies’ strategies and experiences in building HTA considerations into the TPP. Eligible survey participants were the senior management of Global HTA/Market Access Departments at 18 top international pharmaceutical companies.

**Results:** 11 companies responded to the survey. All companies included HTA requirements in TPP development, but the timing and process varied. The key focus of HTA input related to health problems and treatment pathways, clinical efficacy/effectiveness, and safety. Variance of HTA methods and different value frameworks were identified as a challenge for development plans. Stakeholder engagement, such as HTA scientific advice, was used to pressure test the TPP.

**Conclusion:** This research provides insight into current practice and potential opportunities for value-based drug development. It demonstrates the evolution of the TPP to encompass HTA requirements and suggests that the TPP could have a role as an iterative communication tool for use with HTA agencies to enhance an integrated evidence generation plan.

## Introduction

Healthcare systems have been moving towards a value-driven approach. With an aging population and rising healthcare costs, it is vital for decision makers to ascertain where to spend and on whom to spend based on available healthcare budget (Porter, 2009). With the purpose to inform decision making in order to promote an equitable, efficient and value-based health system, health technology assessment (HTA) has emerged and evolved as a multidisciplinary process that uses explicit methods to determine the value of a health technology (O'Rourke et al., 2020). HTA agencies evaluate a (new) health technology such as a medicine based on its relative clinical effectiveness, and/or cost effectiveness to assess if this product provides the best value for money (Rutledge, 2010). However, a range of different methods utilized by HTA agencies may have led to divergent HTA recommendations for pricing and reimbursement, which has resulted in inequitable patient access to new technologies in different jurisdictions (Nicod, 2017). Several studies focusing on the disparity of HTA recommendations have been conducted in the past decade; these have called for improvement of HTA methodology, as well as better collaboration and communication among HTA agencies (Kleijnen et al., 2012; Nicod and Kanavos, 2012; Nicod et al., 2016; Nicod, 2017). The European Network for HTA (EUnetHTA) was set up in 2006 to facilitate HTA collaboration in Europe. A key product of EUnetHTA was the development of the “HTA core model,” a methodological framework to enable international collaboration in producing HTA and efficient sharing of information (Kristensen et al., 2009). The EUnetHTA core model defined a standardized set of HTA questions and contained the following nine domains: current use, technical, safety, clinical effectiveness, cost, and economic evaluation, ethical analysis, organizational aspects, patient and social aspects, and legal aspects (European Network for Health Technology Assessment European Network For Health Technology Assessment, 2016). This value framework has been adapted for production of relative effectiveness assessment (REA) (Kleijnen et al., 2012) for new medicines among European jurisdictions; a recent study evaluating the REA confirmed its benefit in addressing the heterogeneity across HTA agencies and potentially standardizing data requirements (Chassagnol et al., 2020).

In current practice, the submission to HTA agencies for pricing and reimbursement recommendations follows shortly after the regulatory approval; except in Australia and Canada, where companies can submit the HTA dossier during the regulatory review to streamline the timing of the two decision-making processes. Therefore, at the time of the regulatory review and HTA assessment, regulators and HTA agencies use similar data, which are generated from global clinical trials. As a result, companies need to consider not only regulatory requirements during development but also generating evidence that addresses HTA needs. Companies have been refining their internal structures and development strategies to incorporate HTA perspectives into clinical development (Wang et al., 2020). HTA agencies have also started engaging with companies during development to provide early scientific advice. Early scientific advice can either be provided by a single HTA agency, a consortium of multi-HTA agencies, or jointly with a regulator (Wang et al., 2016). Despite efforts by companies and agencies to improve their process and communicate early during development, a key question that remains for companies is how to adapt the requirements from different HTA agencies into a global development plan.

In addition to the HTA evaluation, various value frameworks have emerged in the recent years to assess the value of a new technology. A number of US-oriented value assessment frameworks that are disease-focused have been developed to measure and communicate the value of a new medicine for decision making, such as the American College of Cardiology and the American Heart Association (ACC-AHA) value framework; the Conceptual Framework to Assess the Value of Cancer Treatment Options, developed by the American Society of Clinical Oncology (ASCO); the Institute for Clinical and Economic Review (ICER) Value Framework; the National Comprehensive Cancer Network (NCCN) Evidence Blocks; and the Patient-Perspective Value Framework (PPVF) (Garrison et al., 2018). Notably, ICER has grown its influence over the years to inform payer decisions on funding a new technology (Pizzi, 2016). Hence, companies need to navigate different types of value frameworks during development and run a few scenarios to help understand the value proposition of their products and to ensure the development plan is capturing value-adding components (Neumann et al., 2018).

An essential tool used by companies in the context of planning the clinical development is the target product profile (TPP). The TPP outlines the desired “profile” or characteristics of a target product that is aimed at a particular disease or diseases. There is no defined template for a TPP, however, it is generally structured as a synopsis of its intended labelling. The TPP states the intended use, target populations and other desired attributes of products, including safety and efficacy-related characteristics [World Health Organization (WHO)]. The TPP has been used as an effective communication tool with regulators during drug development and is associated with more efficient regulatory review times (Breder et al., 2017; Tyndall et al., 2017). Many regulatory agencies issue guidance to companies on the development of TPPs (US Food and Drug Administration, 2007; European Medicines Agency, 2009). The World Health Organization (WHO) has also developed TPP documents to inform companies and healthcare decision makers on R&D and public health priorities [World Health Organization (WHO), 2022]. Considering the increasing importance of HTA and other value frameworks in the reimbursement decision, a robust TPP needs to be built to address HTA/payer perspectives, in order to guide an integrated evidence generation plan to aid companies in their development and marketing strategies (Sax et al., 2015). Consequently, companies need to create a dynamic TPP that has a clearly stated value proposition for a new technology. This involves understanding the current standard of care and potential reimbursement environment, navigating through different HTA systems and value frameworks on the evidentiary requirements, and ensuring the right health outcome data is collected during the clinical development phase.

Currently, the concept of the TPP is not commonly used in the context of downstream decision making by HTA agencies. Nevertheless, the TPP has become essential in the upstream decision making by companies and serves as a roadmap for a product’s development and HTA/payer strategy. This study is therefore designed to assess the current practices and experiences of companies in building HTA/payer perspectives into the development plan through the TPP. Specifically, the objectives were to 1) evaluate the challenges faced by companies from different HTA agencies, 2) identify companies’ practices of TPP development that address HTA/payer perspectives, 3) explore companies’ stakeholder engagement strategies during development to test the value proposition.

## Materials and Methods

This study was developed by building on previous Centre for Innovation in Regulatory Science (CIRS) research, which collected quantitative data from pharmaceutical companies on individual products to assess the impact of HTA during drug development and roll out (Wang et al., 2020).

### Study Design

This research was designed in the form of an opinion survey to provide a qualitative assessment of companies’ strategies and experiences in building HTA/payer considerations early into development through the TPP. A pilot questionnaire was developed in September 2019 and reviewed by potential responders from two invited pharmaceutical companies in October 2019. Feedback was provided on the clarity of the questions and was used to finalize the survey on 31 October 2019.

Eligible participants were international pharmaceutical companies with large R&D budgets (2019 budget >1 billion USD), which reflected their innovativeness and value-based medicine development approach. 18 companies were selected based on this purposive sampling, as well as being members of CIRS to ensure the timeliness of the study and maximize the response rate. Questionnaires were sent to the senior management of Global HTA/Market Access Departments at these companies *via* email on 7 November 2019, and they were asked to complete and return the survey by 28 November 2019. Feedback from both the company’s Global HTA Department and local HTA affiliates were gathered and provided as a consolidated survey response to CIRS.

### Structure of the Study Questionnaire

The survey was designed as a cross-sectional questionnaire consisting of eight multiple-choice, closed questions and one open question (Supplementary Appendix). It was organized into three sections: company challenges and solutions for key markets (questions regarding outstanding issues raised by HTA/payers and potential solutions); current practices of companies to build value into the TPP (questions regarding the timing of TPP development, cross-function involvement and HTA/payer perspectives included in the TPP); and company strategies for testing the value proposition during development (questions regarding stakeholder engagement and utilization of relevant value frameworks). A free-text comment option was provided for each question to allow for further clarification. The selection of the HTA agencies in this study was based on the importance of the related market to companies. For the US, where there is no initialized HTA organization, ICER was assessed as a comparator to the HTA agencies and represented an independent value assessment body.

### Data Processing and Analysis

The responses were manually tabulated into a Microsoft Excel file and analyzed using descriptive analysis. Analysis was conducted inductively, data were expressed as absolute number of respondents for each analysis, and ranking was applied where suitable. Free text comments were reviewed and analyzed using the constant comparative method, which involved comparing and contrasting concepts to inform relationships between phrases expressed by the study participants to identify emerging themes (Boeije, 2012). To protect the confidentiality of the individual companies, only aggregated results were presented in this paper.

## Results

11 out of the 18 pharmaceutical companies responded the survey (61% response rate). Nine of the 11 respondents were in the top 25 companies by R&D expenditure in 2019 (Christel, 2019), reflecting the research intensity of the companies and the innovativeness of their development pipelines.

### Understanding Key Health Technology Assessment/Payer Challenges

Firstly, the study assessed the challenges that companies have experienced from key HTA bodies in Australia, Canada, England, France, Germany, Italy, the Netherlands, and ICER in the United States. For each jurisdiction, the respondents were asked to rate three issues frequently raised by the agencies that impact market access decisions. Not all companies provided data for each jurisdiction; results were expressed as the absolute number of responders rating each issue (Table 1).

**TABLE 1:** Outstanding issues that companies have been challenged by HTA/payers on the evidence of a new medicine.

Outstanding issues by area *n* = number of companies who rated this issue		Germany (IQWiG/G-BA) *n* = 9	England (NICE) *n* = 9	France (HAS) *n* = 8	Australia (PBAC) *n* = 8	Canada (CADTH) *n* = 8	United States (ICER) *n* = 7	Italy (AIFA) *n* = 5	Netherlands (ZIN) *n* = 4
Health problem and treatment pathway	Inappropriate patient identification	1	1						
	Inferior place in treatment pathway	1							
Cost-related issues	Not cost-effective/unacceptable price vs. comparator		7		8	7	3	3	4
	Budget impact	1	1	1	2	1	1	2	2
Clinical-related issues	Invalid endpoints	4	1	1	1	2	2	2	
	Comparator not accepted	5	2	2		1	1	2	
	Insufficient efficacy/improvement over comparator	3	3	6	3	4	2	3	2
	Length of trial deemed too short/lack of longer-term outcomes or follow-up	6	5	3	4	5	3	1	1
	Interpretation of external validity of registration trials does not meet local conditions	1	1						
	Inappropriate sub-group selection	1	2	2	3	2	2	1	1
Cost/clinical- related issues	Uncertainty in indirect comparison		1	1	1		2		
Safety	Insufficient safety evidence		1	1	2		1	1	
Patients and social aspects	Insufficient societal benefit								1

In Australia, Canada and England, the most frequently raised issues on the evidence of a new medicine were “not cost-effective,” and “lack of longer-term outcomes.” In Germany and France, where the HTA recommendation is mainly based on added therapeutic value, the outstanding issues centered around comparators, such as insufficient improvement over comparator, comparator choice being unacceptable, the validity of the endpoint and lack of longer-term outcomes or follow-up. In comparison, there was a diversity of issues experienced by companies with ICER in the United States.

### Building Health Technology Assessment/Payer Perspectives Into Target Product Profile Development

**FIGURE 1:**
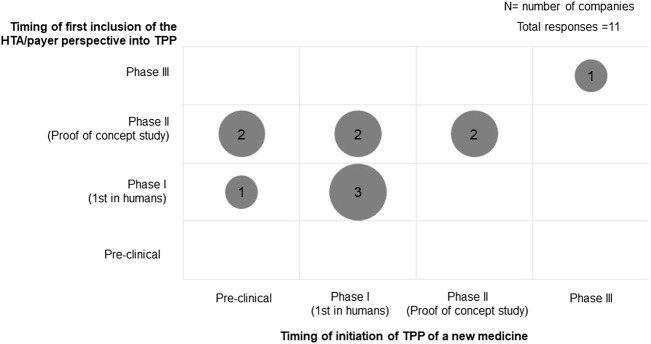
Timing of the initiation of TPP development and inclusion of HTA/payer perspectives.

All participating companies had a TPP to guide the evidence generation plan during drug development. The timing of the initiation of TPP development and the inclusion of HTA/payer perspectives varied among companies (Figure 1). Three companies initiated the TPP during pre-clinical development, while most companies started developing the TPP during Phase I development (5 of 11). HTA/payer perspectives were built into the development plan and were mostly incorporated in the TPP during Phase II (6 of 11). When comparing whether the HTA/payer perspective was included in the TPP since its inception, there was a mix in practices: five companies incorporated HTA/payer perspectives at the beginning of TPP development, whereas six companies included it later. In particular, the companies that started TPP development during the pre-clinical phase did not build in HTA/payer perspectives until Phase I development had started.

**FIGURE 2:**
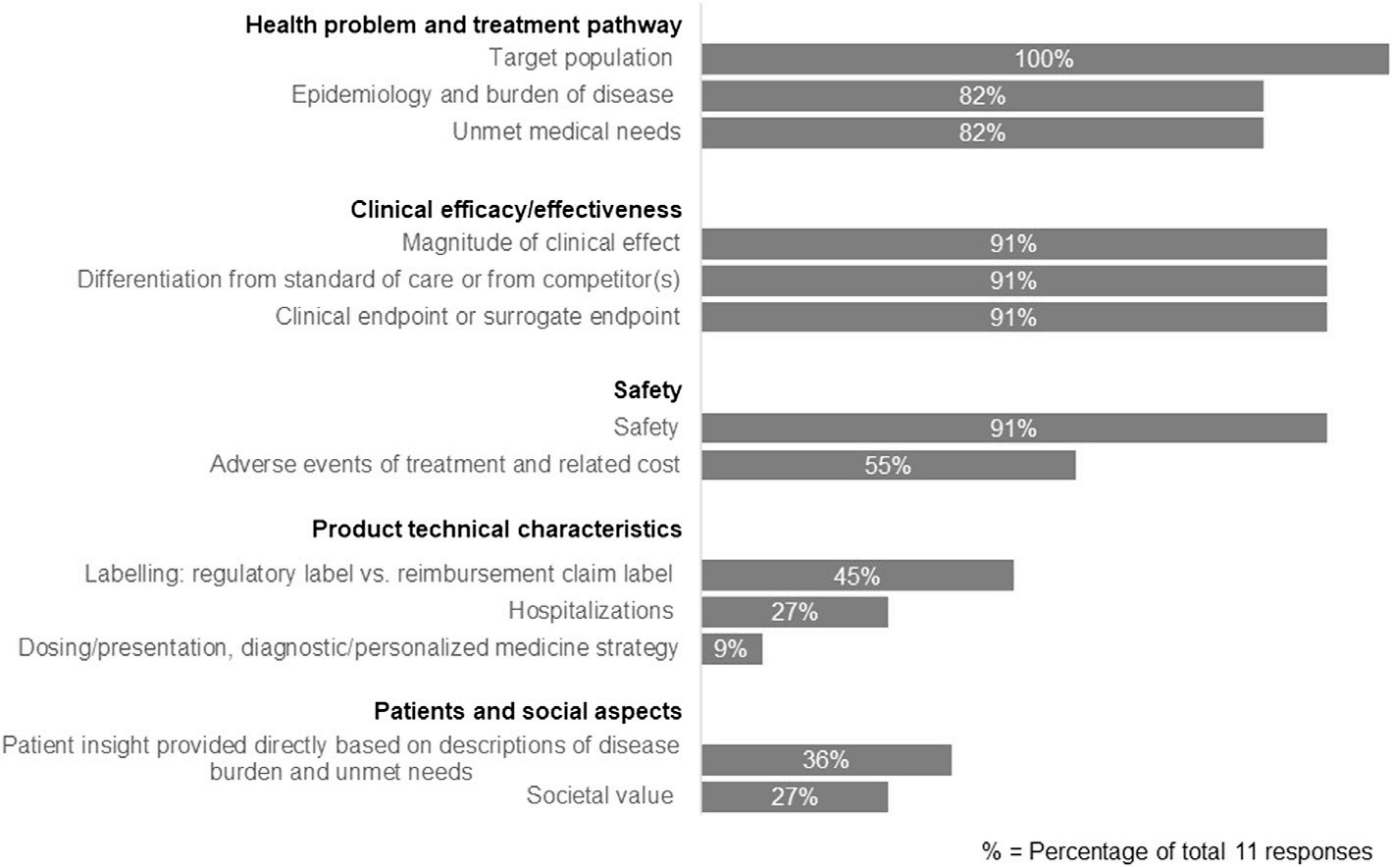
Components included in the TPP that reflect HTA/payer perspectives.

We further assessed the specific components included in the TPP that reflect HTA/payer perspectives (Figure 2). The results showed that companies focused on three main areas: health problem and treatment pathway, clinical efficacy/effectiveness, and safety. More specifically, the components always included in the TPP were on target population (100% companies), safety (91%), magnitude of clinical effect (91%), differentiation from the standard of care or competitors (91%), the clinical endpoint or surrogate endpoint (91%), epidemiology and burden of disease (82%) and unmet medical needs (82%). In addition, hospitalization was rated as a key component (64%) in the TPP development, but this was only considered when necessary to address HTA/payer needs on an ad hoc basis.

**FIGURE 3:**
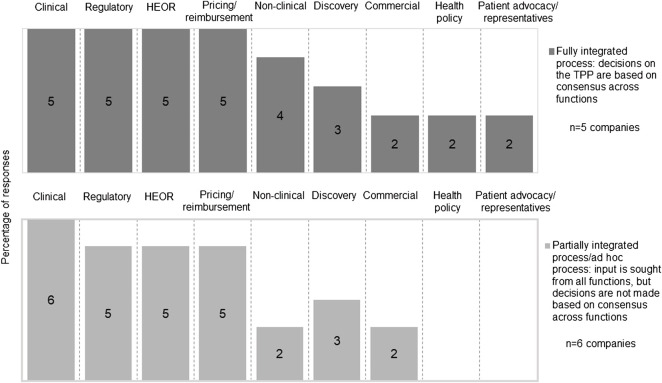
Cross function involvement in the development of the TPP.

The development of a TPP involved multiple functions within a company, however, the process to consolidate the input from different functions was not always systematic. Five companies had a fully integrated approach where TPP decisions were based on consensus across functions, while six companies had a partially integrated process that tended to prioritize regulatory perspectives over HTA/payer perspectives or made the TPP decisions on an ad hoc basis. Clinical, regulatory, health economics and outcomes research (HEOR) and pricing and reimbursement functions were most frequently reported to be involved in TPP development (Figure 3). Two companies reported the participation of a health policy group, and two companies reported the engagement of a patient advocacy group/representative in TPP development; the involvement of these functions was fully integrated.

### Testing Value Propositions With Internal and External Stakeholders

**FIGURE 4:**
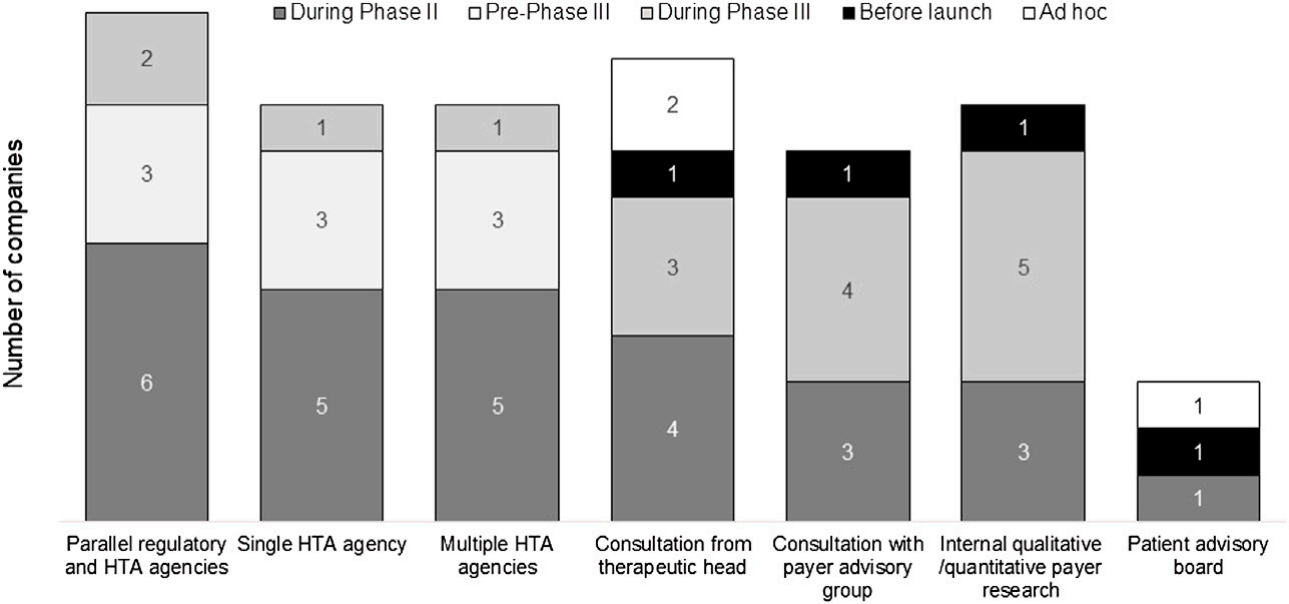
Stakeholder engagement strategy to test the value proposition.

To optimize the TPP of a new medicine, stakeholder engagement was used to “pressure test” the value proposition of the new drug (Figure 4). The survey results showed various internal and external engagement methods utilized by companies, including formal advice from agencies (parallel regulatory-HTA, single HTA, and multiple HTA advice), internal payer research, external payer advisory groups, consultations with therapeutic heads, and patient advisory boards. All companies studied in the survey had experience of internal and external stakeholder engagement. Formal agency advice was usually sought during phase II or pre-phase III, and other types of input tended to occur later in development or on an ad hoc basis.

The majority (10 of 11) of companies also assessed the proposed evidence generation plan for a new medicine against a current value framework in the relevant therapeutic area. The most utilized framework was ICER (60% of responders), followed by PPVF (50%), the European Society for Medical Oncology (ESMO) framework (40%), ASCO (40%), NCCN (40%), ACC/AHA (30%), and EUnetHTA Core Model (20%).

Thematic analysis identified a number of key challenges and potential solutions for building value propositions early into development plans to meet the needs of different jurisdictions (Table 2). These building blocks will be supported by companies’ evolvement of increasing internal awareness of HTA, prioritizing resources, and better alignment internally across multi-functional teams.

**TABLE 2:** Key challenges and potential solutions for building the value proposition sufficiently early in the development program to meet the needs of different jurisdictions.

Practical Challenges	Potential solutions
Limited HTA resource during early development	Raise awareness of the need for HTA resource in early development
Uncertainty in the clinical outcome	Iterative value proposition based on clinical outcome
Internal alignment across functions	Better understanding of the impact of HTA requirements on development to provide incentives for early alignment
Divergent stakeholder needs and priorities	Recognize the impact and make explicit tradeoffs/choices
Stakeholder interaction not early enough	Clear strategy and resource for early advice that can be utilized for development
Changes in treatment/reimbursement landscape	Scenario planning and good competitor intelligence

## Discussion

The TPP is a projection of the expected safety, efficacy/effectiveness and value proposition of a new product and supports companies’ decision making regarding technology design, strategic evidence generation and future marketing strategy. This paper examined the current experiences of pharmaceutical companies in addressing HTA/payer needs through the development of the TPP; the results collected from 11 participating companies provided a unique insight into current operational practice and potential opportunities for value-based drug development.

### Target Product Profile Development That Underpins Companies’ Internal Health Technology Assessment/Payer Strategy

The TPP is developed during early stages of drug development and is typically structured in the format of regulatory labelling; the TPP has been used frequently in communication with regulatory agencies to support market authorization (Tyndall et al., 2017). Our study showed an evolution of TPP development to encompass HTA/payer requirements. All the responding companies indicated that HTA/payer perspectives were included in the TPP. However, we observed a mix of practices in the timing of development of a TPP, with half of respondents starting the TPP development with HTA/payer needs in mind, and the other half including HTA/payer requirements after the TPP was established. Therefore, while the TPP can be established as early as before clinical development, the incorporation of HTA/payer requirements was built in at a later stage, mostly during phase II development. The variation in practice may be related to the involvement of HTA/market access teams in internal cross-functional processes.

Good levels of engagement of clinical, regulatory, HEOR, and pricing and reimbursement teams were observed in TPP development in our study. However, the internal decision-making process was not always fully integrated. Our finding is consistent with one of our earlier studies, which recognized that input from HEOR teams was sought during development, but final decisions were prioritized based on the regulatory requirements (Wang et al., 2018). Respondents recommended ways to improve the internal process, such as raising awareness of the impact and requirements of HTA and prioritizing resources to address HTA needs. A more aligned process with systematic internal decision making will facilitate efficient development of the TPP, and at the same time, a systematically developed TPP can also help to align objectives across different company functions and accelerate development timelines (Lambert, 2010). Two companies also engaged with patient advocacy group/representative in TPP development. With the increasing focus on patient-centered drug development, it would be interesting to assess how patient groups will be further participating in TPP development (Crawford et al., 2017; Kluetz and Bhatnagar, 2021).

Nevertheless, when examining the specific HTA/payer requirements incorporated in the TPP, only 36% respondents stated that “patient insight provided directly based on description of disease burden and unmet needs” was included. HTA/payer considerations included in the TPP concentrated on elements that support the clinical effectiveness evaluation: target population, magnitude of clinical effect, clinical endpoint or surrogate endpoint, safety and differentiation from standard of care. The unmet medical need from the HTA/payer perspective was also included in the TPP by most companies (9 out of 11). Yet, a recent study explored the definition of unmet medical need and concluded that its quantification depended on different stakeholders and their decision context. Therefore, there was a need to align the perspectives on unmet medical need and its measures within the broader value framework for decision making (Vreman et al., 2019). Further development on this topic will be helpful for companies to enhance the TPP with a clear understanding and articulation of unmet medical need.

### Dynamic Target Product Profile Development to Address External Stakeholder Needs

Comparing to the focus on clinical effectiveness in the TPP, our study showed the outstanding issues raised by HTA agencies were mostly “not cost-effective and “unacceptable prices” in Australia, Canada, England, the Netherlands and ICER in the United States. “Lack of longer-term outcomes” and “insufficient improvement over comparators” were reported to be frequent challenges in Germany and France. The outstanding challenges were related to the varying requirements from HTA agencies and how they assess added value in the context of their national healthcare system (Wang et al., 2020). An industry survey pointed out that evidence that supported value proposition at the global level will provide the direction of strategy and key value messages, but then the information must be adapted to the local context, considering variations in standards of care and treatment practices across different markets (Kooreman et al., 2014). In addition, economic value is assessed within the context of national healthcare resources, therefore, jurisdictional pricing and reimbursement strategy will need to be built at the national level (Lucioni and Jommi, 2017). Our study showed that companies have a good understanding of challenges raised by HTA agencies, and the thematic analysis in Table 1 listed the areas of outstanding issues. The learning from jurisdictional experiences will help to improve understanding of HTA/payer needs during development, and an improved TPP during development will in turn facilitate a better evidence generation plan and increase the likelihood of future commercial success. Future studies could concentrate on the impact of the inclusion of the HTA perspective during development on jurisdictional patient access; further indicators can be built based on the value elements included in development, comparing to the added value assessed by HTA agencies. This will be enhanced by the transparency, consistency, and predictability of the HTA decision-making process. In particular, pharmaceutical companies have emphasized transparency as the key principle of value frameworks: transparency in the method and transparency in the types of data and models used (Eddy et al., 2012; Angelis et al., 2020).

HTA agencies have been improving their methodologies and process to ensure a robust and efficient approach to assess the value of a new technology (The National Institute for Health and Care Excellence, 2021). Initiatives are also underway to refine value frameworks; the Professional Society for Health Economics and Outcomes Research (ISPOR) Special Task Force developed a value flower containing 12 elements of value assessment, which expanded beyond traditional clinical and cost evaluation and included elements such as “value of hope” (Lakdawalla et al., 2018). Nevertheless, it is not practical to encompass all value elements or HTA requirements during development. The 2017 HTA International (HTAi) Policy Forum discussed the development of value frameworks used by HTA agencies and third-party organizations and called for agreement and refinement of the core components of value frameworks (Oortwijn et al., 2017).

As companies are creating the TPP prior to Phase II, it will take approximately 4–7 years before the product receives regulatory approval and undergoes subsequent HTA assessment, at which point the evidence requirements and reimbursement environment may have changed. It has been suggested by a company to focus on a core list of elements such as avoidable uncertainty during development and make changes to adapt to HTA needs (Facey et al., 2015). An iterative process leads to the creation of a dynamic TPP document, which will be initially developed focusing on a core list of evidentiary requirements and then be updated as new outcomes are generated from the clinical trial and as the treatment landscape changes.

### Ensuring Target Product Profile Development Through Stakeholder Interactions

A key strategy to test the value proposition of a product is stakeholder engagement. This survey showed that internal activities such as qualitative or quantitative payer research and consultation with the therapeutic head were mostly used, while external advice meetings with HTA agencies and payer advisory groups were frequently sought. Most companies in the study stated that they assessed the proposed evidence generation plan for a new medicine against a current value framework in the relevant therapeutic area. The most utilized value framework was ICER, followed by the PPVF, ESMO, and ASCO frameworks. A study by Wild and colleagues showed that testing the product profile with value attributes will help to identify different scenarios and understand perceived product value (Wild and Mukku, 2011). The EUnetHTA HTA Core Model has also been utilized by companies; it has been viewed as a useful framework to standardize the domain of HTA questions and understand the common terminology (Gyldmark et al., 2018). In addition, one company has developed internal access evidence generation tools based on the HTA Core Model, which has a direct impact on drafting the TPP (Ducournau et al., 2019).

There has been a proliferation of early HTA advice programs in recent years, available at both national and international levels. Our survey showed that the most frequently used format was parallel regulatory-HTA advice. Recent experiences of these advice meetings have been positive, with the benefit of aligning perspectives among different stakeholders and offering opportunities to shape the development plan (Tafuri et al., 2016; Vlachaki et al., 2017; Maignen and Kusel, 2020; Wang et al., 2022). It was acknowledged that although the role, function and remit of regulatory and HTA agencies are different and should remain distinct, more interactions and alignment between the agencies will be helpful to ensure more efficient drug development. Potential interactions between regulatory and HTA agencies have been suggested to converge clinical requirements, align national review and reimbursement process, and increase transparency and trust between stakeholders (Centre for Innovation in Regulatory Science, 2021). A previous study also suggested that payers should be involved in TPP development, which can facilitate evidence generation and understanding of payer related issues and unmet medical needs (Fatoye et al., 2019). Nevertheless, the advice provided by HTA agencies is non-binding and the treatment and reimbursement landscape may change by the time the product reaches market access; therefore, internal activities are also critical to enable good competitor intelligence and scenario planning.

Companies participated in early scientific advice meetings where HTA agencies generally used a briefing book to summarize the key characteristics of a product, and the key questions to be discussed at the meeting. Although the TPP has been frequently used in early advice meetings with regulators (Tyndall et al., 2017), it was unknown how the TPP has facilitated the development of the briefing book for HTA advice, and how the advice taken from HTA agencies has been built into the dynamic TPP. As a development tool, it would be useful for the TPP to be used not only internally by companies, but also as an iterative communication tool with regulators, HTA, payers and patient groups to enhance an integrated evidence generation plan.

### Strengths and Limitations of the Study

Our findings should be interpreted in light of this study’s strengths and limitations. This paper is based on a perception survey from 11 participating companies therefore the results reflect the view of those companies from purposeful sampling. However, the participants represent international companies that are focusing on development of innovative medicine, therefore are a good marker of HTA practices. For each question in the survey, not all of the participants responded due to their experiences and perceptions; analyses were therefore shown with both absolute numbers and percentages. In addition, the HTA perspectives in the paper were assessed from companies’ positions. Further study on the topic could be explored from HTA/payer perspectives to provide a balanced view on how best to build HTA into a sufficient development and roll out process.

## Conclusion

The TPP has been used as a blueprint to guide companies on their development plan for a new medicine. In this study, all participating companies have included HTA/payer perspectives in TPP development. However, there were practical divergencies in terms of the timing of the inclusion, the cross-functional process and the key requirements included. It showed that companies were at different levels of utilizing the TPP in drug development to address future HTA/payer needs. Considering the variance of HTA methods and different value frameworks used in assessing the value of a new technology, a dynamic TPP is essential to facilitate evidence generation plans by focusing on a core list of components, which can be pressure tested through early scientific advice with agencies, payer research and internal assessment against relevant value frameworks. Building on this paper, further research could explore the wider application of the TPP, such as in supporting communication with HTA agencies or payers.

## Data Availability

The original contributions presented in the study are included in the article/Supplementary Material, further inquiries can be directed to the corresponding author.

## Abbreviations

ACC, american college of cardiology; AHA, american heart association; AIFA, agenzia italiana del farmaco; ASCO, american society of clinical oncology; CADTH, canadian agency for drugs and technology in health; CIRS, center for innovation in regulatory science; ESMO, european society for medical oncology; EUnetHTA, european network for health technology assessment; G-BA, gemeinsamer bundesausschuss; HAS, haute autorité de santé; HEOR, health economics and outcomes research; HTA, health technology assessment; ICER, the institute for clinical and economic review; IQWiG, institutfür qualität und wirtschaftlichkeit im gesundheitswesen; NCCN, national comprehensive cancer network; NICE, national institute for health and care excellence; PBAC, pharmaceutical benefits advisory committee; PPVF, avalere/faster cures patient-perspective value framework; TPP, target product profile; ZIN, zorginstituut nederland.
