# Supplementary material for: Challenges and Opportunities for Companies to Build HTA/Payer Perspectives Into Drug Development Through the Use of a Dynamic Target Product Profile
Source: Front Pharmacol. 2022 Jul 18;13:948161. doi: 10.3389/fphar.2022.948161 (PMC9340272; doi:10.3389/fphar.2022.948161)
Supplement: Supplementary file 1 [file DataSheet1.DOCX]

1. **Incorporating value proposition into the development of TPP**

**(Target Product Profiles)**

1. **Please choose one option by putting an X for the following questions**

| 1. **When does your company first start the development of TPP of a new medicine?**   Select one by putting an X | 1. **When does your company first include the HTA/payer perspective into the TPP to demonstrate the value proposition?**   Select one by putting an X |
| --- | --- |
| Global project frame  Pre-clinical development  Phase I (1^st^ in humans)  Phase II (PoC study)  Start in Phase IIb  Start in Phase III  Before regulatory submission | Global project frame  Pre-clinical development  Phase I (1^st^ in humans)  Phase II (PoC study)  Start in Phase IIb  Start in Phase III  Before regulatory submission |

1. **Which functions within your company are involved in the process of building the TPP for a new medicine?** Select all applicable options by putting an X.

Discovery

Non-clinical

Clinical

Regulatory

HEOR

Pricing and reimbursement

Healthcare Policy

Patient advocacy group/patient representatives

Others, please specify __________________­_

1. **Is there a systematic process to consolidate input from different functions into the development of the TPP?**

Yes, fully integrated process : Decisions on the TPP are based on consensus across functions

Yes, partially integrated process: Input is sought from all functions, but regulatory perspective is prioritized over HTA/payer perspectives

No: Decisions are made on an ad hoc basis

Others, please specify ____________________________________________

1. **What are the elements that your company includes in the TPP that reflect HTA/payer perspectives?**

| **Elements in the TPP that reflect HTA/payer perspectives** | **Included in TPP all the time** | **Considered but only included on an ad hoc basis** |
| --- | --- | --- |
| Unmet medical needs |  |  |
| Epidemiology and burden of disease |  |  |
| Target population |  |  |
| Differentiation from standard of care or from competitor(s) |  |  |
| Clinical endpoint or surrogate endpoint |  |  |
| Magnitude of clinical effect |  |  |
| Safety |  |  |
| Hospitalizations |  |  |
| Adverse events of treatment and related cost |  |  |
| Labelling: regulatory label vs. reimbursement claim label |  |  |
| Patient insight provided directly based on descriptions of disease and treatment burden and unmet needs |  |  |
| Societal value |  |  |
| Others :Please specify |  |  |

1. **“Pressure testing” the value proposition of a new medicine**
2. **What is your company’s strategy for testing the value proposition of a new medicine during development?** Select all applicable options by putting an X.

| **Strategy** | **Timing of interaction**  *(please provide the phase of drug development)* |
| --- | --- |
| Seek early scientific advice from a single HTA agency |  |
| Seek early scientific advice from multiple HTA agencies |  |
| Seek early scientific advice from parallel Regulatory and HTA agencies |  |
| Consultation with payer advisory group |  |
| Consultation from therapeutic head |  |
| Internal qualitative /quantitative payer research |  |
| Patient advisory boards |  |
| Others, please specify |  |

1. **Does your company assess the proposed evidence generation plan for a new medicine against any value framework in the relevant therapeutic area?**

| **Value assessment framework** | Select all applicable options by putting an X. |
| --- | --- |
| The European Society for Medical Oncology (ESMO) |  |
| The Institute for Clinical and Economic Review (ICER) |  |
| The American College of Cardiology/American Heart Association (ACC/AHA) frameworks |  |
| The American Society of Clinical Oncology (ASCO) |  |
| The National Comprehensive Cancer Network (NCCN) framework |  |
| The Avalere/FasterCures Patient-Perspective Value Framework (PPVF) |  |
| Others, Please specify |  |

1. **Value interpretation during roll-out at key jurisdictions**

**8. What are the top 3 outstanding issues that your company has been challenged by HTA/payers on the evidence of a new medicine?**

Please select top 3 issues for each jurisdiction from the list on below

| 1. *Invalid endpoints* 2. *Comparator not accepted* 3. *Insufficient improvement over comparator* 4. *Insufficient efficacy* 5. *Insufficient safety evidence* 6. *Length of trial deemed too short* 7. *Lack of longer term outcomes or follow-up* 8. *Interpretation of external validity of registration trials does not meet local conditions* 9. *Inappropriate patient identification* 10. *Inappropriate sub-group selection* 11. *Inferior place in treatment pathway* 12. *Not cost-effective* 13. *Unacceptable price vs. comparator* 14. *Budget impact* 15. *Insufficient societal benefit* 16. *Others (please specify in the table below)* |
| --- |

| **Jurisdictional HTA** | **Top 3 outstanding issues that were frequently raised by HTA that have an impact on the market access**  **(***Please select relevant letters from the list above)* |
| --- | --- |
| Australia (PBAC) |  |
| Canada (CADTH) |  |
| England (NICE) |  |
| France (HAS) |  |
| Germany (IQWIG/G-BA) |  |
| Italy (AIFA) |  |
| Netherlands (ZIN) |  |
| US (ICER) |  |

1. **What were the key internal barriers for building the value proposition sufficiently early into the development programme to meet the needs of the different jurisdictions?**

Please list the top three challenges and potential solutions

| **Challenges** | **Solutions** |
| --- | --- |
|  | 1. |
|  | 2. |
|  | 3. |

1. **Do you have any comments you would like to provide with regard to this topic that you believe would be of value to discuss at the upcoming CIRS Technical forum? Please specify.**

|  |
| --- |

*Please sign and date:*

| Company | Name |
| --- | --- |
| Department | Date |

Thank you for completing this questionnaire!

Please return to [twang@cirsci.org](mailto:twang@cirsci.org) by 20^th^ November
